# The relationship between 3S (Seiri, Seiton, and Seiso) behaviors, and psychological distress and work engagement

**DOI:** 10.3389/fpubh.2025.1646180

**Published:** 2025-10-03

**Authors:** Tomoko Sawajima, Tomohisa Nagata, Kiminori Odagami, Takahiro Mori, Nuri Purwito Adi, Koji Mori

**Affiliations:** ^1^Department of Occupational Health Practice and Management, Institute of Industrial Ecological Sciences, University of Occupational and Environmental Health, Kitakyushu, Japan; ^2^Tokyo Health Care Office, Health Care Center, Central Japan Railway Company, Tokyo, Japan; ^3^Department of Community Medicine, Faculty of Medicine, Universitas Indonesia, Jakarta, Indonesia

**Keywords:** three S, Seiri, Seiton, Seiso, psychological distress, work engagement

## Abstract

**Objectives:**

This study aimed to examine the relationship between 3S [Seiri (Sort), Seiton (Set), and Seiso (Shine)] behaviors in the workplace and workers’ psychological distress and work engagement.

**Methods:**

A prospective cohort study was conducted using an online survey among workers in Japan (*n* = 9,451 at baseline; *n* = 6,156 at follow-up). At baseline, participants were asked whether they routinely practiced 3S behaviors in the workplace every day. Psychological distress was measured using the Japanese version of Kessler 6-Item Psychological Distress Scale (K6), and work engagement was assessed using the Japanese version of the Utrecht Work Engagement Scale (UWES-9) at baseline and follow-up. Covariates included industry type and K6/UWES-9 at baseline. Multiple regression analyses were conducted to evaluate the relationship between 3S behaviors at baseline and K6/UWES-9 at follow-up.

**Results:**

There were 3,862 (62.7%) workers who practiced 3S behaviors. Workers who did not practice 3S behaviors had significantly higher psychological distress than those who did (standardized coefficient = 0.03, *p* = 0.006). There was no statistical difference in work engagement between workers who practiced 3S behaviors and those who did not (standardized coefficient = −0.01, *p* = 0.339).

**Conclusion:**

Daily practice of 3S behaviors was related to lower psychological distress among workers, suggesting that 3S may serve not only as a tool for quality management but also as a potential strategy for improving mental health in the workplace.

## 1 Introduction

Three S (3S)—Seiri (Sort in English), Seiton (Set in English), and Seiso (Shine in English)—are reported to have been practiced in Japanese companies since the 1800s (1). “Seiri” means classifying things into necessary and unnecessary items and removing the unnecessary ones. “Seiton” means organizing necessary items so that they can be easily accessed. “Seiso” means to sweep and tidy up. Adding “Seiketsu (Standardize in English)” and “Shitsuke (Sustain in English)” to this, it is sometimes called Five S (5S). These practices have helped companies overcome various problems such as lack of workers discipline and diligence, waste reduction and savings during economic downturns, workplace safety, and productivity improvement.

Although 3S is an activity that originated in Japan, it is now used as a quality management tool in manufacturing and healthcare services in other countries (2). Many companies widely implement 3S behaviors, often as part of occupational health and safety initiatives. It is recommended to work on organizing and setting things in order as part of the prevention of accidents involving falls. In addition, 3S is included in workplace mental health action checklists aimed at improving the work environment, and is expected to have positive psychological effects on workers (3).

Such psychological effects are often assessed using both negative and positive indicators, with psychological distress and work engagement being commonly used indicators. Psychological distress is commonly defined as a state of emotional suffering characterized by symptoms of depression and anxiety, often accompanied by somatic complaints such as insomnia or fatigue. It is widely conceptualized as a continuum of symptom severity rather than a discrete diagnostic category (4). In contrast, work engagement is generally understood as a positive, fulfilling work-related state of mind that is characterized by vigor, dedication, and absorption. Vigor reflects high levels of energy and resilience at work, dedication denotes strong involvement and a sense of enthusiasm and significance, and absorption describes being fully concentrated and happily engrossed in work (5).

3S behaviors, based on the Job Demands-Resources (JD-R) Model, have the potential to reduce psychological distress and increase work engagement. The JD-R model, a widely used framework of occupational stress research, outlines two primary processes (6, 7). The first is the motivational process, in which job resources—defined as physical, psychological, social, or organizational aspects of the job that help achieve work goals, reduce job demands and their associated costs, or promote personal growth and development—enhance motivation, including work engagement. The second is the health-impairment process, in which job demands—defined as aspects of the job that require sustained physical or mental effort and are therefore associated with certain physiological and psychological costs—lead to strain, such as psychological distress. Job resources play a critical role in buffering the negative effects of job demands, thereby contributing to employees’ health and performance. When the 3S of work are in good condition, it is believed that employees have a clearer understanding of what tasks need to be done and how to perform them. Such a work environment with good 3S conditions can itself serve as a job resource. Specifically, 3S improves role clarity, reduces unnecessary effort, and enhances efficiency. In this way, good 3S conditions are expected to lower perceived job demands and simplify work processes, thereby decreasing workload and the likelihood of errors. As a result, good 3S status may be associated with lower psychological distress. Moreover, since job resources are positively associated with work engagement, good 3S conditions may also contribute to higher work engagement.

Previous research suggests that a cluttered environment can reduce feelings of fulfillment, happiness and the sense of safety and security derived from being in one’s personal space (8). 3S behaviors improve a cluttered environment, which in turn may increase feelings of safety and security. In addition, the psychological effects of tidy-up behavior on university students reported positive effects on wellbeing (9). However, there are no studies that have identified the relationship between 3S behaviors and negative mental health status such as psychological distress, or positive work-related psychology such as work engagement. This study aims to clarify the relationship between 3S behaviors status, psychological distress, and work engagement in the workplace.

## 2 Methods

### 2.1 Study design and participants

This is a prospective cohort study conducted through an online survey, forming part of the Work, Well-being and Safety for Occupational health practice and management II Study (W2S-Ohpm II Study). The baseline survey conducted in March 2023, and the follow-up survey was conducted in December 2023. This study was approved by the Ethics Committee of the University of Occupational and Environmental Health, Japan (Approval numbers: R4-077). All participants provided their informed consent through an online form available on the survey website.

The target population for the survey was registered monitors of Rakuten Insight, Inc. (Tokyo, Japan). Rakuten Insight, Inc. managed the survey operations and communicated to the registered monitors that a certain number of points would be awarded for responding to the questions. At the time of the survey, 497,760 people were registered, and some of those who were working were asked to participate in the study. We set the target sample size to 10,000 participants. The eligible population for the survey was workers in Japan aged 20 or older at the baseline survey. Sampling was conducted considering sex, age, and prefecture (geographic region) based on the actual Japanese workforce to ensure the target population accurately represented workers in Japan. Among 21,965 registered monitors who answered the initial screening questions, 10,000 had matched the survey’s criteria (worker status, sex, age, and region). We analyzed 9,451 respondents after excluding 549 who were judged to have given invalid response. The criteria for the invalid response are as follows: unusually height and/or weight; respondents who indicated that they were engaged in work for 0 days or 0 h; respondents who worked more than 150 h/wk.; respondents who provided the same responses for total weekly working hours including overtime hours and for only weekly overtime hours; respondents who answered unusually long 1-way commuting times of 7 h or more; and respondents who stated that they had 16 or more family members living with them. Detailed sampling methods are reported in a previous study (10). A follow-up survey was conducted in December 2023 for these subjects.

Since we conducted the survey online, there are no missing values in the questionnaire (we set it so that an error occurs when the field is left blank).

### 2.2 Assessment of 3S behaviors status

We asked the participants, “Do you routinely practice 3S (Seiri, Seiton, and Seiso) in your workplace every day?” Participants answered “yes,” “no,” or “unknown” to the question. To the best of our knowledge, no validated questionnaire has been found for assessing 3S behaviors. However, many Japanese people are familiar with the term 3S, and the words “Seiri, Seiton, and Seiso” are commonly used. In this study, the phrase “every day” was added to the questionnaire to assess whether 3S behaviors were practiced habitually.

### 2.3 Outcomes

We set two outcomes at the follow-up survey: psychological distress and work engagement. We evaluated psychological distress using the Japanese version of Kessler 6-Item Psychological Distress Scale (K6) (11), and work engagement using the nine-item Japanese version of the Utrecht Work Engagement Scale (UWES-9) (12).

The Japanese version of the K6 has been translated and validated, and its screening performance has been confirmed to be comparable to that of the original version. The K6 is a nonspecific measure of psychological distress consisting of six questions asking participants if they had felt nervous, hopeless, restless, or fidgety during the past 30 days; so depressed that nothing could cheer you up; that everything was an effort; and worthless. Participants were asked to respond to each of the items using a 5-point scale that ranged from 1 (all of the time) to 5 (none of the time) as their state over the past month. Responses were then reverse-coded (0–4), with higher scores indicated more psychological distress (range: 0–24). The Cronbach’s alpha for K6 in this survey was 0.92.

The nine-item Japanese version of the Utrecht Work Engagement Scale (UWES-9) has previously been translated into Japanese, and the Japanese version has been shown to have acceptable internal consistency and reliability, as well as factor and construct validity. The UWES-9 includes measures of vigor (three item), dedication (three item), and absorption (three item), with each item measured on a seven-point response scale ranging from 0 (never) to 6 (always/every day). Overall scores on the UWES-9 are calculated by averaging the scores of each item (range: 0–6). The Cronbach’s alpha for UWES-9 in this survey was 0.95.

### 2.4 Covariates

The covariates included industry category and K6/UWES-9 at the baseline survey. In Japan, 3S initiatives in the workplace are often implemented as part of occupational accidents and/or injuries prevention measures and are common in manufacturing and construction industries. To eliminate the influence of industry type, industry type was added as a covariate. The industry types were classified into 20 categories based on the Japan’s standard industrial classification (13). The baseline K6 scores or UWES-9 scores were also added as covariates. The reason for this was to exclude the possibility that the original psychological distress or work engagement influenced 3S behaviors and to more strongly demonstrate temporal relevance.

### 2.5 Statistical analysis

We calculated the number and proportion of workers who implemented 3S behaviors by sex, age, and industry. We verified the relationship between each category and the presence or absence of 3S behaviors using a chi-square test.

We conducted multiple regression analyses to examine the relationship between 3S behaviors status and psychological distress (K6 score) as well as work engagement (UWES-9 scores) at follow-up. For psychological distress, we used two models: Model 1 was adjusted for industry type; Model 2 was adjusted for industry type and baseline K6 scores. Similarly, for work engagement, Model 1 was adjusted for industry type; Model 2 was adjusted for industry type and baseline UWES-9 scores.

The 3S behaviors status was assessed using three options: “yes,” “no,” or “unknown.” The questions used to assess the 3S behaviors status in this study have not been validated. Therefore, as a sensitivity analysis, we conducted two additional analyses: one including “unknown” as “yes” and another including ‘unknown’ as “no.”

We defined statistical significance as a two-tailed *p*-value of less than 0.05. All analyses were conducted using Stata Statistical Software (Release 19.5; StataCorp LLC, College Station, TX, United States).

## 3 Results

Among 9,451 participants, a total of 6,156 individuals participated in the follow-up survey (response rate was 65%). The characteristics of the study participants are shown in Table 1. The workers with 3S behaviors were 3,862 (62.7%), without 3S behaviors were 1,848 (30.0%) and unknown workers were 446 (7.2%). The mean age (standard deviation [SD]) of each 3S behaviors status (“with 3S behaviors,” “without 3S behaviors,” “unknown”) were 48.2 (SD: 13.1), 47.7 (SD: 12.9), and 45.1 (SD: 13.4), respectively. The K6 scores at follow-up were 4.6 (5.0), 5.4 (5.5), and 5.9 (5.7), while the UWES-9 scores at follow-up were 2.6 (1.2), 2.3 (1.1), and 2.2 (1.1), respectively.

**Table 1:** Characteristics of participants in this study by 3S behaviors status.

Characteristics		Worker with 3S behavior	Worker without 3S behavior	Unknown
N		3,862	1848	446
Age, mean (SD)		48.2 (13.1)	47.7 (12.9)	45.1 (13.4)
Sex, n (%)				
	Men	2,145 (55.5%)	1,202 (65.0%)	263 (59.0%)
	Women	1717 (44.5%)	646 (35.0%)	183 (41.0%)
Industry category, n (%)				
	Agriculture and forestry	33 (0.9%)	31 (1.7%)	5 (1.1%)
	Fisheries	2 (0.1%)	1 (0.1%)	1 (0.2%)
	Mining and quarrying of stone and gravel	4 (0.1%)	4 (0.2%)	0 (0.0%)
	Construction	204 (5.3%)	76 (4.1%)	18 (4.0%)
	Manufacturing	683 (17.7%)	278 (15.0%)	61 (13.7%)
	Electricity, gas, heat supply and water	58 (1.5%)	27 (1.5%)	5 (1.1%)
	Information and communications	151 (3.9%)	142 (7.7%)	27 (6.1%)
	Transport and postal services	157 (4.1%)	90 (4.9%)	19 (4.3%)
	Wholesale and retail trade	427 (11.1%)	177 (9.6%)	51 (11.4%)
	Finance and insurance	140 (3.6%)	82 (4.4%)	16 (3.6%)
	Real estate and goods rental and leasing	86 (2.2%)	57 (3.1%)	11 (2.5%)
	Scientific research, professional and technical services	82 (2.1%)	53 (2.9%)	7 (1.6%)
	Accommodations, eating and drinking services	135 (3.5%)	20 (1.1%)	20 (4.5%)
	Living-related and personal services and amusement services	94 (2.4%)	42 (2.3%)	5 (1.1%)
	Education and learning support	272 (7.0%)	118 (6.4%)	21 (4.7%)
	Medical, health care and welfare	591 (15.3%)	171 (9.3%)	47 (10.5%)
	Compound services	35 (0.9%)	18 (1.0%)	4 (0.9%)
	Services	367 (9.5%)	223 (12.1%)	64 (14.3%)
	Public sector	229 (5.9%)	139 (7.5%)	28 (6.3%)
	Unlabeled	112 (2.9%)	99 (5.4%)	36 (8.1%)
K6 at follow-up survey (range 0–24), mean (SD)		4.6 (5.0)	5.4 (5.5)	5.9 (5.7)
WE at follow-up survey (range 0–6), mean (SD)		2.6 (1.2)	2.3 (1.1)	2.2 (1.1)

3S, Seiri, Seiton and Seiso; SD, standard deviation; UWES, Utrecht Work Engagement Scale.

Table 2 shows the number and proportion of workers implementing 3S behaviors. While 67% of female workers habitually performed 3S behaviors, only 59% of male workers did so. The implementation rate of 3S behavior increased with age. Compared to manufacturing (67%) and construction (69%), implementation rates were low in primary industries and information and communications (47%), while high in accommodations, eating and drinking services (77%) and medical, health care and welfare (73%).

**Table 2:** Number and proportion of workers implementing 3S behaviors.

Characteristics		N	%	*p*-value^†^
Sex				<0.001
	Men	2,145	59.4	
	Women	1717	67.4	
Ageclass				0.022
	20–29	459	60.4	
	30–39	602	59.2	
	40–49	965	64.2	
	50–59	897	62.6	
	60–69	803	64.5	
	70-	136	68.7	
Industry category				<0.001
	Agriculture and forestry	33	47.8	
	Fisheries	2	50.0	
	Mining and quarrying of stone and gravel	4	50.0	
	Construction	204	68.5	
	Manufacturing	683	66.8	
	Electricity, gas, heat supply and water	58	64.4	
	Information and communications	151	47.2	
	Transport and postal services	157	59.0	
	Wholesale and retail trade	427	65.2	
	Finance and insurance	140	58.8	
	Real estate and goods rental and leasing	86	55.8	
	Scientific research, professional and technical services	82	57.7	
	Accommodations, eating and drinking services	135	77.1	
	Living-related and personal services and amusement services	94	66.7	
	Education and learning support	272	66.2	
	Medical, health care and welfare	591	73.1	
	Compound services	35	61.4	
	Services	367	56.1	
	Public sector	229	57.8	
	Unlabeled	112	45.3	

3S, Seiri, Seiton and Seiso. ^†^Chi-square test.

Table 3 shows the relationship between 3S behaviors and psychological distress/work engagement at follow-up. After adjusting for industry type (Model 1), psychological distress of workers without 3S behaviors was higher compared to workers with 3S behaviors (standerdized coefficient = 0.03, *p* = 0.006). In Model 2 (further included baseline K6 scores as covariates), psychological distress of workers without 3S behaviors was higher (standerdized coefficient = 0.03, *p* = 0.006).

**Table 3:** The relationship between 3S behaviors and psychological distress and work engagement at the follow-up survey.

Outcome	Model 1			Model 2		
	β	B (95%CI)	*p*-value	β	B (95%CI)	*p*-value
Outcome: Psychological distress						
Worker with 3S behavior	Ref.	Ref.		Ref.	Ref.	
Worker without 3S behavior	0.07	0.82 (0.53 to 1.12)	<0.001	0.03	0.29 (0.08 to 0.50)	0.006
Unknown	0.06	1.24 (0.73 to 1.75)	<0.001	0.02	0.48 (0.12 to 0.85)	0.010
Outcome: Work engagement						
Worker with 3S behavior	Ref.	Ref.		Ref.	Ref.	
Worker without 3S behavior	−0.10	−0.26 (−0.32 to −0.19)	<0.001	−0.01	−0.02 (−0.07 to 0.02)	0.339
Unknown	−0.08	−0.37 (−0.49 to −0.25)	<0.001	−0.01	−0.04 (−0.12 to 0.04)	0.296

Model 1, Adjusted for industry category; Model 2, Adjusted for industry category and K6 score (outcome = psychological distress) or WE score (outcome = WE) at the baseline survey; 3S, Seiri, Seiton and Seiso; SE, standard error; WE, work engagement; B, unstadnardized coefficient; β, standardized coefficient.

Work engagement of workers without 3S behaviors was lower compared to workers with 3S behaviors in Model 1 (standerdized coefficient = −0.10, *p* < 0.001). In Model 2 (further included baseline UWES-9 scores as covariates), there was no statistically significant relationship between 3S behaviors and work engagement (standerdized coefficient = −0.01, *p* = 0.339).

The results of the additional analysis that included “unknown” as either “no” or “yes” for the 3S behavior are shown in Supplementary Tables 1, 2. The group of workers without 3S behavior/unknown had significantly higher K6 values compared to the group of workers with 3S behavior (Supplementary Table 1). The group of workers without 3S behavior also showed a similar trend toward higher K6 values compared to the group of workers with 3S behavior/unknown (Supplementary Table 2). WE did not show statistically significant differences, while consistent with the results of the main analysis.

## 4 Discussion

Workers who practiced 3S behaviors had significantly lower psychological distress than those who reported not practicing in 3S behaviors. There was no statistically significant relationship between 3S behavior and work engagement.

In this study, working in a cluttered environment was suggested to induce feelings of depression and anxiety. It has been shown that when people set goals and work on something, they retrieve the information they need from what they have in their field of vision (14). In a disorganized and cluttered environment, they are likely to spend more effort looking for the information they need (15). This may lead to the individual perceiving their workload as high. Based on the JD-R model, it is considered that depression and anxiety (assessed by K6) increased as a stress response. Additionally, a previous study examining the psychological effects of tidy-up behavior of university students reported positive effects on wellbeing from tidy-up behavior (9). An organized and neatened environment may reduce the amount of effort required to obtain the necessary information for a goal, and also reduce discomfort with a cluttered environment due to tidiness.

3S behaviors and work engagement were not related. According to the JD-R model (7, 8), work engagement is associated with work and personal resources. In the analysis without adjusting for work engagement at the baseline survey, there was a relationship between 3S behaviors and work engagement. This analysis suggests that individuals with high work engagement may be more likely to engage in 3S behaviors habitually. This is likely based on the fact that no significant relationship was found between 3S behaviors and work engagement at the follow-up survey after adjusting for work engagement at the baseline survey. There may be no effect of 3S behaviors on increasing work engagement. This point requires further clarification through intervention studies.

Companies have addressed organizational challenges such as fostering discipline, diligence, promoting savings, and ensuring safety through 3S. In addition, the Mental Health Action Checklist for improving the workplace environment, published by the Ministry of Health, Labour and Welfare, has a 30-item action framework, including an organizing item on “making each individual’s work area easier to work in Ministry of Internal Affairs and Communications (16).” The study suggests that the 3S may reduce depression and anxiety among workers. This finding indicates that 3S behaviors are related with lower psychological distress, suggesting that in the workplace, it is expected to promote initiatives within companies not only as a tool for quality management but also as one of the efforts aimed at improving workers’ mental health.

In this study, we have not been able to examine the motivations behind the participants’ 3S behaviors, specifically whether these behaviors were voluntary or based on workplace rules. If the results of this survey indicate effects due to spontaneous behavior, it is possible that some elements of the 3S behaviors influence mental health. Conversely, if the behaviors are based on workplace rules, there may be factors influencing mental health not only related to the 3S behaviors themselves but also to the context in which these behaviors occur. We believe it is necessary to confirm this in future research.

The implementation rate of the 3S behaviors varies depending on attributes, which should be taken into consideration when intervening in the workplace. In this study, the implementation rate of 3S behaviors was low among men and younger individuals. This suggests that daily lifestyle habits are related to the implementation of 3S behaviors. Additionally, this study revealed that there are variations in implementation rates depending on industry. This suggests that workplace initiatives influence 3S behaviors. In accommodation and food services, implementing 3S is considered an essential task and is therefore incorporated into daily operations. In construction, manufacturing, and medical and welfare services, 3S behaviors are often considered essential habits for performing daily tasks without mistakes, and are carried out as activities that everyone in the workplace participates in Anggarini (17). On the other hand, 3S behaviors are not progressing in the information and communications industry. The reason for this may be that the industry mainly involves computer work, and even if 3S is not thoroughly implemented for files stored inside computers, it is unlikely to be noticed by others. In summary, both private life and personal characteristics, as well as workplace initiatives, influence 3S behaviors.

This study has several limitations. First, the questions regarding the presence or absence of 3S behaviors have not been confirmed for validity and reliability. In Japan, children are exposed to the term “3S” from elementary school. There is a daily habit of all students and teachers working together on the 3S in the classroom after lunch break. Therefore, rather than breaking down each behaviors of the 3S, we asked about 3S behavior as a series of actions. However, there may have been people who did not recognize what the 3S behaviors were. In response to the question, “Do you practice the 3S every day?,” 446 (7.2%) participants answered “unknown.” This is likely because the definition of “3S” in the question was ambiguous. We conducted an additional analysis (sensitivity analysis) to see how the results would differ when people who answered “unknown” were allocated to the group that performed 3S behaviors and the group that did not. The results did not differ significantly from the main results (Supplementary Tables 1, 2). It is necessary to consider methods for identifying 3S behaviors in the future. Second, this study only covers workers who are registered with an internet survey company. There is a possibility that people who do not normally use the internet are not included, which limits the generalizability of this study.

## 5 Conclusion

Companies have historically addressed various challenges through 3S practices. The results of this study suggest that workers who practice 3S behaviors in the workplace experience lower levels of psychological distress. Sharing these findings with both workers and managerial staff may help to reaffirm the importance of 3S practices in the workplace. Since this study cannot clarify the mechanism by which 3S behavior is associated with low psychological distress, further research is needed to elucidate the mechanism.

## Data Availability

The original contributions presented in the study are included in the article/Supplementary material, further inquiries can be directed to the corresponding author.
