# Supplementary material for: The relationship between 3S (Seiri, Seiton, and Seiso) behaviors, and psychological distress and work engagement
Source: Front Public Health. 2025 Oct 3;13:1646180. doi: 10.3389/fpubh.2025.1646180 (PMC12532780; doi:10.3389/fpubh.2025.1646180)
Supplement: Supplementary file 2 [file Table_2.docx]

Supplemental Table 2. The relationship between 3S behavior and psychological distress/work engagement at the follow-up survey

|  |  | Multivariate adjusted | | |
| --- | --- | --- | --- | --- |
|  |  | β | B (95%CI) | p value |
| Outcome: Psychological distress | | | | |
|  | Worker with 3S behavior/Unknown | ref. | ref. |  |
|  | Worker without 3S behavior | 0.02 | 0.24 (0.04 to 0.45) | 0.022 |
|  |  |  |  |  |
| Outcome: Work engagement | | | | |
|  | Worker with 3S behavior/Unknown | ref. | ref. |  |
|  | Worker without 3S behavior | -0.01 | -0.02 (-0.06 to 0.03) | 0.444 |

Multivariate-adjusted model was adjusted industry category and K6 score (outcome=psychological distress) or work engagement score (outcome= work engagement) at the baseline survey

3S: Seiri, Seiton and Seiso

B, unstadnardized coefficient; β, standardized coefficient
